# Amplicon-Based Next-Generation Sequencing as a Diagnostic Tool for the Detection of Phylotypes of *Cutibacterium acnes* in Orthopedic Implant-Associated Infections

**DOI:** 10.3389/fmicb.2022.866893

**Published:** 2022-04-07

**Authors:** Diana Salomi Ponraj, Jeppe Lange, Thomas Falstie-Jensen, Nis Pedersen Jørgensen, Christen Ravn, Anja Poehlein, Holger Brüggemann

**Affiliations:** ^1^Department of Clinical Medicine, Aarhus University, Aarhus, Denmark; ^2^Department of Orthopedic Surgery, Horsens Regional Hospital, Horsens, Denmark; ^3^Department of Orthopedic Surgery, Aarhus University Hospital, Aarhus, Denmark; ^4^Department of Infectious Diseases, Aarhus University Hospital, Aarhus, Denmark; ^5^Department of Orthopedic Surgery, Lillebaelt Hospital, Kolding, Denmark; ^6^Department of Genomic and Applied Microbiology, Institute of Microbiology and Genetics, University of Göttingen, Göttingen, Germany; ^7^Department of Biomedicine, Aarhus University, Aarhus, Denmark

**Keywords:** *Cutibacterium acnes*, orthopedic implant-associated infections, prosthetic joint infections, single-locus sequence typing, amplicon-based next-generation sequencing, sonication fluid

## Abstract

The diagnosis of orthopedic implant-associated infections (OIAIs) caused by the slow-growing anaerobic bacterium *Cutibacterium acnes* is challenging. The mild clinical presentations of this low-virulent bacterium along with its ubiquitous presence on human skin and human-dominated environments often make it difficult to differentiate true infection from contamination. Previous studies have applied *C. acnes* phylotyping as a potential avenue to distinguish contamination from infection; several studies reported a prevalence of phylotypes IB [corresponding to type H in the single-locus sequence typing (SLST) scheme] and II (SLST type K) in OIAIs, while a few others found phylotype IA_1_ (more specifically SLST type A) to be abundant. However, phylotype determination has mainly been done in a culture-dependent manner on randomly selected *C. acnes* isolates. Here, we used a culture-independent amplicon-based next-generation sequencing (aNGS) approach to determine the presence and relative abundances of *C. acnes* phylotypes in clinical OIAI specimens. As amplicon, the SLST target was used, a genomic fragment that is present in all *C. acnes* strains known to date. The aNGS approach was applied to 30 sonication fluid (SF) samples obtained from implants removed during revision surgeries, including 17 *C. acnes* culture-positive and 13 culture-negative SF specimens. In 53% of the culture-positive samples, SLST types were identified: relative abundances were highest for K-type *C. acnes*, followed by H- and D-type *C. acnes*. Other types, including A- and C-type *C. acnes* that are more prevalent on human skin, had low relative abundances. The aNGS results were compared with, and confirmed by a culture-dependent approach, which included the isolation, whole genome sequencing (WGS) and phylotyping of 36 strains of *C. acnes* obtained from these SF samples. Besides serving as a powerful adjunct to identify *C. acnes* phylotypes, the aNGS approach could also distinguish mono- from heterotypic infections, i.e., infections caused by more than one phylotype of *C. acnes*: in eight out of nine culture-positive SF samples multiple *C. acnes* types were detected. We propose that the aNGS approach, along with the patient’s clinical information, tissue and SF cultures and WGS, could help differentiate *C. acnes* contamination from true infection.

## Introduction

*Cutibacterium acnes* (*C. acnes*, formerly known as *Propionibacterium acnes*) is a slow-growing Gram-positive anaerobic bacterium (SGAB) that is found preferentially in the pilosebaceous glands of the human skin (McDowell et al., 2013; Aubin et al., 2014). It is increasingly being reported from various implant-associated infections, including orthopedic implant-associated infections (OIAI; Portillo et al., 2013; Achermann et al., 2014; Aubin et al., 2014; Ponraj et al., 2021). It is most frequently isolated from prosthetic joint infections (PJI) of the shoulder, but has also been implicated in OIAI of spine, hip, knee, elbow, and other joints (Sampedro et al., 2009; Bacle et al., 2017; Renz et al., 2018a; Namdari et al., 2019).

The diagnosis of *C. acnes* OIAI is not easy. The mild clinical presentation, muted immune responses to this low-virulent organism and the lack of a gold standard make the diagnosis challenging (Renz et al., 2018a; Pruijn et al., 2021). The ubiquitous nature of *C. acnes* on human skin and human-dominated environments increases the risk of contamination of diagnostic specimens during surgery and/or specimen processing. Some studies reported the recovery of *C. acnes* from deep tissues during primary surgery despite antibiotic prophylaxis and skin preparation with chlorhexidine and isopropyl alcohol (Matsen et al., 2015; Torrens et al., 2019). Moreover, *C. acnes* have been detected intracellularly within stromal cells and macrophages of intraarticular tissue samples of patients undergoing primary shoulder surgery, suggesting the possibility of it being an intraarticular commensal (Hudek et al., 2021). Thus, when *C. acnes* is isolated in patients without obvious clinical or laboratory features of OIAI, it is difficult to determine its clinical significance, and if it should be treated (Dorrestijn and Pruijn, 2021; Falstie-Jensen et al., 2021; Neufeld et al., 2021; Pruijn et al., 2021; Spek et al., 2021). There is therefore a risk of over- or undertreatment.

*Cutibacterium acnes* is a multiphyletic species with six distinct phylogenetic lineages, namely IA_1_, IA_2_, IB, IC, II, and III, based on multi-locus sequence typing (MLST) that has been applied in culture-dependent studies (McDowell et al., 2005, 2012; Kilian et al., 2012). In addition, to determine the population structure of *C. acnes* in culture-dependent and culture-independent studies, a single-locus sequence typing (SLST) scheme based on core genome phylogeny was developed that can distinguish 10 main SLST types (Scholz et al., 2014). The SLST types A–E represent phylotype IA_1_ and SLST types F–L correspond to phylotypes IA_2_, IC, IB, II, and III, respectively. Previous attempts to distinguish infection and contamination based on *C. acnes* phylotyping yielded conflicting results. In several studies, *C. acnes* strains of types IB (SLST type H) and II (SLST type K) were found to be overrepresented in suspected cases of OIAI (Sampedro et al., 2009; McDowell et al., 2013; Aubin et al., 2017; Salar-Vidal et al., 2021), while other studies found phylotype IA_1_ (in particular SLST type A and also SLST type D) to be more prevalent (El Sayed et al., 2019; Liew-Littorin et al., 2019). On the skin surface, strains of phylotype IA are usually predominant (McDowell et al., 2013; McLaughlin et al., 2019; Paetzold et al., 2019).

This study was conducted to evaluate the possibility of using a culture-independent phylotyping method on clinical specimens to identify OIAI-causing *C. acnes* phylotypes, namely amplicon-based next-generation sequencing (aNGS) using the SLST fragment as amplicon target. The method is based on the SLST amplicon sequencing approach developed by Scholz et al. (2014). The method was subsequently adapted for Illumina sequencing (Paetzold et al., 2019). Recently, a slightly modified approach, also adapted for Illumina sequencing, was used to determine the *C. acnes* phylotype distribution on skin of patients undergoing primary and revision shoulder arthroplasty surgeries (Hsu et al., 2020a). In addition, the study aimed at the distinction between monotypic infections, i.e., infections due to a single *C. acnes* phylotype only, and heterotypic infections. Previous studies, based on *C. acnes* cultivation and subsequent random selection of colonies, have suggested that *C. acnes* might be able to cause heterotypic infections (El Sayed et al., 2019; Bumgarner et al., 2020).

Here, implants removed during revision surgeries were collected and sonicated to maximize bacterial recovery. The obtained sonication fluid (SF) samples were cultured, and all *C. acnes* isolates were phylotyped and genome-sequenced. In addition, SF samples from 30 implants (17 culture-positive for *C. acnes* and 13 culture-negative) were subjected to aNGS to determine the presence and relative abundance of all *C. acnes* SLST types. A high relative abundance of strains belonging to H- and K-type *C. acnes* was found; these were detected in heterotypic infections. In addition, based on the combined evaluation of clinical and molecular methods our study suggests that only a fraction of *C. acnes* culture-positive cases represent true OIAIs.

## Materials and Methods

### Study Design, Setting, and Participants

Patients who underwent revision surgery between August 2019 and September 2020 at the Department of Orthopedic Surgery, Aarhus University Hospital, were included in the study. The removed orthopedic implants were collected, irrespective of the anatomic location (shoulder, knee, hip, elbow, and ankle), type of implant (plates, screws, liners, nails, and prostheses), or indication for revision (hardware irritation, dislocation of implant, suspected, or overt infection). Processing of all implants was performed at the Department of Biomedicine, Aarhus University. *A priori*, it was decided to include 100 implants in the study. At the discretion of the surgeons, for some patients, tissue samples for culture were sent to the Department of Clinical Microbiology, Aarhus University Hospital. Tissue culture results, when available, were accessed from the patient’s records. They were considered positive, if two or more of the five tissue cultures showed growth of the same bacteria. Informed consent was obtained from 85 patients to access data from the patient’s record. The study was registered with Region Midtjylland with reference number 661624. The Central Denmark Region ethical committee waived the need for ethical approval. Patient data were collected and managed using REDCap electronic data capture tools hosted at Aarhus University (Harris et al., 2019).

### Collection, Storage, and Transportation of Implants

Orthopedic implants from patients included in the study were collected in the operating room (OR) during revision surgery performed under standard aseptic protocols for implant-associated surgical procedures, and directly placed in sterile, single-use, air-tight, plastic containers (LocknLock, South Korea) by the scrub nurse. If more than one implant was extracted from the same surgical site, they were placed in the same container. The implant was covered at least 90% with sterile saline before the container was sealed in the OR. The sealed containers were stored for maximum about 2 h at room temperature and at 4°C from 2 h to maximum about 24 h until retrieval and implant processing. All implants were subsequently processed by the same person.

### Sonication of Implants

The implants were processed by a previously described vortex-sonication method (Borens et al., 2013). In brief, the method included the following steps: 30 s of vigorous shaking of the sealed container with the implant and sterile saline, followed by 60 s of sonication at 100% power (BactoSonic, Bandelin electronic, Berlin, Germany) and another 30 s of vigorous shaking. Opening of the sealed containers and all further processing of SF were done inside a biological safety class II cabinet (ScanLaf class 2 cabinets: Mars, LaboGene) using standard biosafety level-2 laboratory practices. Separate sterile pipettes were used to harvest SF to two sterile tubes for use in culture-dependent and culture-independent investigations, respectively.

### Cultivation From Sonication Fluid

For culture-dependent analysis, aliquots of 100 μl of SF were inoculated on three different agar media: sheep blood agar (BA), fastidious anaerobic agar (FAA) with horse blood (Thermo Scientific™), and reinforced clostridial agar (RCA; Oxoid, Thermo Scientific™). In addition, the remaining SF was centrifuged at 10,000 *g* (rotor—F 13–14 × 50 Cy, Thermo Scientific™ Sorvall™ RC 6 Plus Centrifuge) for 20 min at 4°C. The pellet was resuspended in 1 ml remaining supernatant and aliquots of 10 and 1 μl of the resuspended pellet were inoculated on BA, FAA, and RCA plates. The plates were incubated anaerobically (Whitley A35 anaerobic workstation) for up to 28 days. The plates were checked for visible microbial growth daily in the first week and thereafter weekly. One additional BA plate inoculated with 100 μl of uncentrifuged SF was incubated aerobically for up to 3 days.

### Colony-Forming Unit Count

In case of visible bacterial growth, the colony-forming unit count (CFU/ml SF) was determined for each colony morphology type, since different colony morphologies could represent different *C. acnes* phylotypes. As per the latest guidelines of The European Bone and Joint Infection Society (EBJIS), growth of >50 CFU/ml from uncentrifuged SF and >200 CFU/ml from centrifuged SF was considered significant (McNally et al., 2021).

### Identification of Bacterial Species by 16S rRNA Sequencing

Bacterial colonies were subcultured on the same media as used for primary growth. Crude extract of bacterial DNA was prepared as previously described (Scholz et al., 2014). 16S rRNA gene amplification was performed using the B4 and B5 primers that amplify the V1–V3 region (Mikkelsen et al., 2000). A PCR reaction mixture of 25 μl containing 8 μl sterile PCR grade water, 2 μl primer mix (5 μmol each of B4 and B5 primers), 5 μl of the 1:100 diluted DNA, and 10 μl of 5Prime Hotmaster mix (Quanta Bio) was prepared. The following thermocycling scheme was used: 94°C for 5 min (1 cycle), 94°C for 1 min, 60°C for 1 min (30 cycles), 72°C for 2 min, and 72°C for 8 min (1 cycle). The PCR products were verified on agarose gels. Sequencing of the PCR products using the B4 and B5 primers was done at Eurofins Genomics (Ebersberg, Germany), and sequence comparison with the NR database at NCBI was done using blastn. A sequence identity >99% of the amplicon sequence with a database entry led to species assignment.

### DNA Isolation and Whole Genome Sequencing of *Cutibacterium acnes*

For genomic DNA extraction of *C. acnes* strains, the MasterPure™ Gram-Positive DNA Purification Kit (Lucigen) was used as per manufacturer’s instructions. Concentration and purity of the isolated DNA were first checked with a NanoDrop ND-1000 (Peqlab, Erlangen, Germany); concentrations were determined using the Qubit® dsDNA HS Assay Kit as recommended by the manufacturer (Life Technologies GmbH, Darmstadt, Germany). Illumina shotgun libraries were prepared using the Nextera XT DNA Sample Preparation Kit and subsequently sequenced on a MiSeq system using the v3 reagent kit with 600 cycles (Illumina, San Diego, CA, United States) as recommended by the manufacturer. Quality filtering was done with version 0.36 of Trimmomatic (Bolger et al., 2014). Assembly was performed with version 3.13.0 of the SPAdes genome assembler software (Bankevich et al., 2012). Version 2.2.1 of Qualimap was used to validate the assembly and determine the sequence coverage (García-Alcalde et al., 2012). In total, 36 *C. acnes* genomes were sequenced with a genome coverage of 31- to 225-fold (in average 139-fold), and all genome sequences were deposited in GenBank.

### Genome Sequence Data Analyses

Gene prediction and annotation of all genomes were performed with PGAP (Tatusova et al., 2016). For phylogenomic analyses, the core genome was identified and aligned with the Parsnp program from the Harvest software package (Treangen et al., 2014). All *C. acnes* genomes available from GenBank (status October 2021, *n* = 286) were used along with the 36 *C. acnes* genomes from this study to build a core genome-based phylogeny. Reliable core genome single-nucleotide variants (SNVs) identified by Parsnp were used for the reconstruction of genome-based phylogeny. Phylogenetic trees were visualized using the Interactive Tree Of Life (Letunic and Bork, 2021). The SLST type assignment based on the genome sequence was done using the SLST assignment tool on http://medbac.dk/slst/pacnes.

### Culture-Independent Determination of the *Cutibacterium acnes* Population

For culture-independent analysis, 30 SF specimens were selected. They included all 17 samples that showed *C. acnes* growth in SF culture, as well as 13 SF specimens that were randomly chosen from implants that were SF culture-negative for *C. acnes*.

#### Sample Preparation and DNA Extraction

About 40 ml of SF per implant that was stored at −20°C until processing was thawed overnight at 4°C and then processed as follows: concentration of SF was done by centrifugation at 15,000 × *g* (rotor—F 13–14 × 50 Cy, Thermo Scientific™ Sorvall™ RC 6 Plus Centrifuge) for 1 h at 16°C. All but approximately 1 ml of the supernatant was discarded, and the pellet was resuspended in the remaining supernatant. DNA was extracted using the DNeasy PowerSoil Kit (QIAGEN, Hilden, Germany) following the manufacturer’s protocol. DNA concentrations were measured using the Qubit dsDNA HS Assay (ThermoFisher Scientific, Waltham, MA, United States) with a Qubit fluorometer following the manufacturer’s instructions.

#### Amplification of the *Cutibacterium acnes* SLST Amplicon Fragment

The SLST fragment was amplified using the primers: 5′-TTGCTCGCAACTGCAAGCA-3′ and 5′-CCGGCTGGCAAATGAGGCAT-3′. PCR reaction mixtures were made in a total volume of 25 μl and comprised 5 μl of DNA sample, 2.5 μl AccuPrime PCR Buffer II (Invitrogen, Waltham, MA, United States), 1.5 μl of each primer (10 μM; DNA Technology, Risskov, Denmark), 0.15 μl AccuPrime Taq DNA Polymerase High Fidelity (Invitrogen, Waltham, MA, United States), and 14.35 μl of PCR grade water. The PCR reaction was performed using the following cycle conditions: initial denaturation at 94°C for 2 min, 35 cycles of denaturation at 94°C for 20 s, annealing at 55°C for 30 s, elongation at 68°C for 1 min, and final elongation step at 72°C for 5 min. PCR products were verified on an agarose gel and purified using the Qiagen Generead™ Size Selection kit (Qiagen, Hilden, Germany). The concentration of the purified PCR products was measured with a NanoDrop 2000 spectrophotometer (ThermoFisher Scientific, Waltham, MA, United States).

#### Amplicon-Based Next-Generation Sequencing

PCR products were used to attach indices and Illumina sequencing adapters using the Nextera XT Index kit (Illumina, San Diego, CA, United States). Index PCR was performed using 5 μl of template PCR product, 2.5 μl of each index primer, 12.5 μl of 2x KAPA HiFi HotStart ReadyMix, and 2.5 μl PCR grade water. The thermal cycling scheme was as follows: 95°C for 3 min, 8 cycles of 30 s at 95°C, 30 s at 55°C, and 30 s at 72°C, and a final extension at 72°C for 5 min. Quantification of the products was performed using the Quant-iT dsDNA HS assay kit (ThermoFisher Scientific, Waltham, MA, United States) and a Qubit fluorometer, following the manufacturer’s instructions. MagSi-NGS^PREP^ Plus Magnetic beads (Steinbrenner Laborsysteme GmbH, Wiesenbach, Germany) were used for purification of the indexed products as recommended by the manufacturer, and normalization was performed using the Janus Automated Workstation from Perkin Elmer (Perkin Elmer, Waltham, MA, United States). Sequencing was conducted using an Illumina MiSeq platform with dual indexing and the MiSeq reagent kit v3 (600 cycles), as recommended by the manufacturer.

#### Bioinformatics

FASTQ sequences obtained after demultiplexing the reads and trimming the primers (Cutadapt v. 3.7; Martin, 2011) were imported into QIIME2 (v. 2021.2; Bolyen et al., 2019). Sequences with an average quality score lower than 20 or containing unresolved nucleotides were removed from the dataset. The paired-end reads were denoised and chimeras removed with DADA2 *via* q2-dada2 (Callahan et al., 2016), and a feature table was generated. These features were then clustered with VSEARCH (Rognes et al., 2016) using q2-vsearch (Rideout et al., 2014) at a threshold of 99% identity against the SLST allele database (accessible *via*: http://medbac.dk/slst/ pacnes: December 2021). Figures were prepared in R (v. 4.0.5) with the packages ggplot2 (v. 3.3.3) and gplots (v. 3.1.1).

## Results

### Patient Material and Implant Processing Workflow

A total of 100 implants from 99 patients were collected for this study. Shoulder prostheses were the most common implants, followed by knee and hip prostheses. In addition, plates and screws from these joints as well as elbow and ankle joints were also included in the study. The location and type of implants included in the study are shown in Supplementary Figure S1A. For 85 patients, we had access to their clinical files. The median age of these patients was 70 years (range 26–91 years) and majority of them were female (61.2%). Aseptic loosening (20.0%), aseptic failure (17.7%) and pain from plates/screws (14.1%) were the three most common reasons as defined by the surgeon responsible for implant removal, while OIAI or suspicion of OIAI was the reason for implant removal in 10.6%. Periprosthetic tissue biopsy samples were sent for culture to the Department of Clinical Microbiology at Aarhus University Hospital in 69 of 85 patients (81.2%), of whom 16 (23.2%) were tissue culture-positive. The demographic and clinical data of these patients are given in Supplementary Figure S1B.

**Figure 1:**
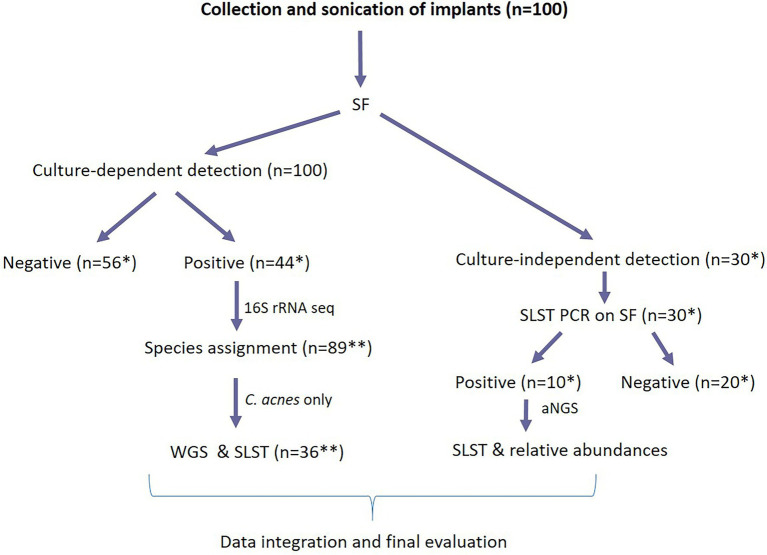
Illustration of the workflow regarding 100 implants processed in this study. Hundred implants were first processed using the culture-dependent method. After obtaining these results, 30 SF specimens were selected (SF from 17 *C. acnes* culture-positive and 13 culture-negative implants) for culture-independent analysis. The final evaluation was based on the integration of patients’ clinical features, periprosthetic tissue culture results and results from the culture-dependent and culture-independent detection in SF. *: number of implants; **: number of bacterial isolates. SF, sonication fluid; WGS, whole genome sequencing; SLST, single-locus sequence typing; aNGS, amplicon-based next-generation sequencing.

All 100 removed implants were processed by the vortex-sonication method (Borens et al., 2013), and the resultant SF of the implants was subjected to culture-dependent and culture-independent analyses. An overview of the workflow and applied analyses is given in Figure 1.

### Culture-Dependent Analysis

Sonication fluid was cultivated under aerobic and anaerobic conditions on three different agar media. Visible bacterial growth was seen in SF from 44 implants (44%). Multiple colony morphology types, either on the same or different agar media were noted in SF culture of 26 implants. In total, 89 bacterial isolates were obtained from 44 culture-positive implants. All bacterial isolates were assigned to species level by 16S rRNA gene sequencing (Supplementary Table S1). In total, 42 isolates were assigned to *C. acnes*, derived from 21 implants; 10 of these implants showed growth of *C. acnes* isolates with more than one colony morphology. CFU counts were determined for each colony morphology type. Based on the EBJIS cut-off values (McNally et al., 2021), 23 of the 42 *C. acnes* isolates (54.8%) had significant CFU counts.

### *Cutibacterium acnes* Whole Genome Sequencing, Single-Locus Sequence Typing, and Single-Nucleotide Variant Analysis

To investigate the *C. acnes* isolates in more detail, genome sequencing and SLST type assignment of the 42 isolates were performed. WGS data analysis revealed that five isolates from four implants had to be reclassified as *Cutibacterium modestum* (data not shown). This shows that the applied species assignment method, relying on 16S rRNA gene sequencing, is not optimal to differentiate *C. acnes* from *C. modestum*, as previously noted (Goldenberger et al., 2021). The remaining 36 *C. acnes* isolates from 17 implants were used in subsequent analyses. The details of the 36 *C. acnes* genomes, including size, coverage, and contig numbers as well as their SLST types are given in Supplementary Table S2.

**Figure 2:**
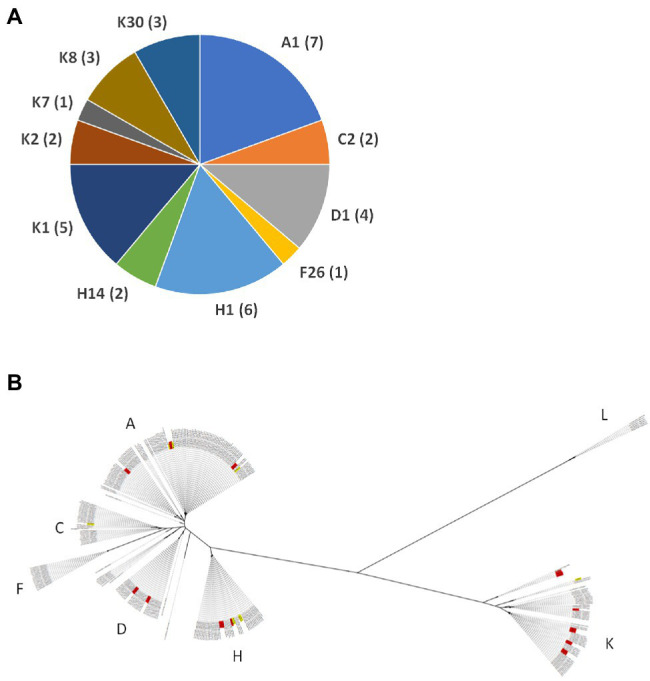
Assignment of SLST types and core genome-based phylogeny of *C. acnes* isolates obtained in this study. **(A)** The SLST types of 36 *C. acnes* isolates are shown. Phylotype II (SLST type K) strains were most often found (39%), followed by strains of phylotypes IA_1_ (SLST types A, C, D; 36%), IB (SLST type H; 22%), and IA_2_ (SLST type F; 3%). **(B)** The core genome-based phylogeny of *C. acnes* is shown, using all genomes available at GenBank (*n* = 286; status October 2021) and the 36 *C. acnes* genomes sequenced here (labeled in red). The SLST types of the main phylogenetic clades are given as letters. A high-resolution version of the figure is available as Supplementary Figure S2.

The SLST type assignment for the 36 strains revealed that 14 isolates belonged to the K type (five K1, three K8, three K30, two K2, and one K7), eight isolates to the H type (six H1 and two H14), seven to A1, four to D1, two to C2, and one to F26 (Figure 2A). Multiple SLST types of *C. acnes* were isolated from five implants. Two implants harbored three SLST types each: K2, K1, and D1 in one implant and K8, H1, and K1 in another implant. Three implants were associated with two SLST types each: K1 and A1, A1 and K7, and H1 and D1, respectively.

Next, a phylogenetic analysis was conducted, based on SNVs in the aligned core genome. The phylogenetic tree showed the expected clustering of strains according to their determined SLST types (Figure 2B; Supplementary Figure S2). There was no obvious separation of the strains based on the anatomical location or type of implant. The analysis revealed that several strains were found to be clonal (Table 1); clonal strains were defined by less than 30 core genome-located SNVs. As expected, most clonal strains were from the same implant but isolated on different agar media. However, also two (SLST type) C2 strains, isolated from two different implants, were clonal. Similarly, four A1 strains were found to be clonal, even though they were all isolated from different implants. This raises the possibility that these C2 and A1 strains are potential contaminants that were acquired during specimen processing.

**Table 1:** Sequenced *Cutibacterium acnes* strains with clonal structure based on numbers of single-nucleotide variants (SNVs).

Strain name	SLST type	SNVs	Origin
SASDk57A, SASDk57B, and SASDk57C	K30	0	One implant, different agar media
HASDk23A, HASDk23B	H14	0	One implant, different agar media
EPSSDk41A, EPSSDk41B	K1	0	One implant, different agar media
SASDk73A, SASDk73D	A1	0	One implant, different agar media
PSSDk50A, PSSDk50D	K2	3	One implant, different agar media
SASDk24A, SASDk24B	H1	12	One implant, different agar media
EASDk81B, EASDk81E, and EASDk81D	K8	1, 22	One implant, different agar media
HASDk1A, KASDk20A	C2	9	Two different implants—possible contaminants
SASDk78B, SASDk4A, KPSSDk45A, and (EPSSDk41C)	A1	4, 7, (66)	Four different implants—possible contaminants

### Culture-Independent Analysis: Single-Locus Sequence Typing PCR and Amplicon-Based Next-Generation Sequencing

**Figure 3:**
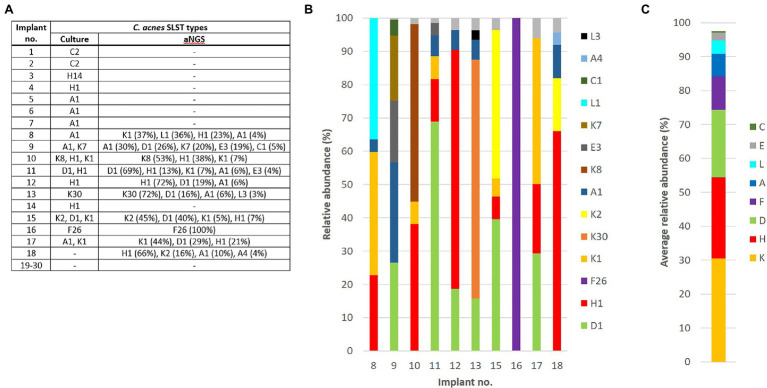
Results of the culture-independent aNGS analysis on 30 sonication fluid specimens. **(A)** Around 30 samples were analyzed by aNGS, including 17 and 13 *C. acnes* culture-positive and -negative implants, respectively. The detected SLST types of the *C. acnes* strains obtained from the 17 culture-positive implants are listed. The SF of 10 implants was amplicon PCR-positive, the respective SLST types determined by aNGS are listed (relative abundances in %). **(B)** The relative abundances of all detected SLST types are shown from the 10 PCR-positive SF samples that were subjected to aNGS and subsequent SLST type assignment. **(C)** The average relative abundance for the main SLST types, determined by aNGS, was calculated across the 10 samples, showing that overall K-type *C. acnes* had the highest relative abundance, followed by H- and D-type *C. acnes*.

Next, we applied a previously developed aNGS approach that can detect all SLST types of *C. acnes* in a given sample and determine their relative abundances without the need for cultivation (Scholz et al., 2014). The first step of this approach is a PCR on DNA extracted from SF to amplify the SLST fragment, a genomic fragment that is present in all strains of *C. acnes* known to date. Around 30 SF specimens were selected, including all 17 samples that exhibited growth of *C. acnes* in SF culture, as well as 13 SF specimens that were randomly chosen from implants that were SF culture-negative for *C. acnes*. All but one of the 13 culture-negative implants (implants no. 18–30) were also negative for the SLST PCR, indicating that these culture-negative samples usually do not contain uncultivatable *C. acnes* or free DNA derived from *C. acnes* (Figure 3A).

Only nine out of the 17 culture-positive implants were positive in the SLST PCR (53%). All amplicons were sequenced and the sequencing data statistics are given in Supplementary Table S3. SLST types H1 and D1 (average abundances 28.8 and 33.1%, respectively) were detected in six implants, while types A1 and K1 (average abundances 10.5 and 19.9%, respectively) were detected in five implants. The other SLST types were less frequently detected (Figure 3B). Summarizing the data according to the main phylotypes, K-type *C. acnes* (phylotype II) had in average the highest relative abundance (30.5%), followed by H-type (IB; 23.9%), D-type (IA_1_; 19.8%), F-type (IA_2_; 9.9%), A-type (IA_1_; 6.6%), and L-type *C. acnes* (III; 3.9%; Figure 3C).

Surprisingly, multiple SLST types were detected in all but one of the nine SF specimens that were PCR-positive, indicating that heterotypic infections might be common. In contrast, in the culture-dependent approach, multiple SLST types were isolated in only five of these nine implants. Regarding these five samples, the predominant SLST type detected by aNGS was also detected by SF culture and subsequent WGS, confirming that the culture-dependent and culture-independent methods are complementary (Figure 3A).

Eight out of the 17 *C. acnes* culture-positive implants were SLST PCR-negative (47%). This indicates that in these cases, *C. acnes* bacteria might have contaminated the sample after implant sonication, e.g., in the cultivation or incubation step. The SLST types of the *C. acnes* isolates in these eight implants included three A1, two H1, two C2, and one H14 strain (Figure 3A). Only one culture-negative implant was positive by SLST PCR (implant no. 18). This could be due to the presence of a strain that is difficult to culture or a potential contamination during the SLST PCR.

### Origin of the Clonal Strains

To further study the origin of the clonal strains and to assess if they could be derived from contamination, skin swabs for cultivation were taken from the person who processed the implants. Colonies that resembled *C. acnes* were isolated and SLST type assignment was done for 10 selected colonies. Strains belonging to the SLST types A1 and C2 were obtained and further genome-sequenced. Genome comparison of these strains with the C2- and A1-type strains obtained from several implants (no. 1, 2, 5, 6, 7, 8, and 17) showed that the A1- and C2-type *C. acnes* strains were clonal, as judged from the low number of SNVs in the core genome (data not shown). This strongly suggests that these A1 and C2 strains were indeed contaminants.

### Evaluation of Significance of *Cutibacterium acnes* Isolation Based on Combined Analyses

All obtained data of the 17 implants that were SF culture-positive for *C. acnes*, i.e., the details of the patients’ clinical features, SF and periprosthetic tissue culture results and aNGS results were combined and analyzed (Supplementary Table S4). Based on the combined data, we categorized the patient cases with the 17 implants in three groups (Table 2).

**Table 2:** Group 1: Infection unlikely, Group 2: Infection likely, and Group 3: Undetermined, respectively.

No.	Joint	Tissue culture results	Sonication fluid—culture-dependent					Sonication fluid—culture-independent	
			Poly-microbial	Strain name	Days to growth	CFU/ml	SLST type	SLST PCR	SLST types from aNGS
**Table 2A: Group 1: Infection unlikely**									
1	Hip	No growth	with *S. capitis*	HASDk1A	6	50	C2	−	−
2	Knee	No growth	No	KASDk20A	21	>250	C2	−	−
3	Hip	No growth	No	HASDk23A/HASDk23B	7	20	H14	−	−
4	Shoulder	No growth	No	SASDk40A	21	20	H1	−	−
5	Shoulder	No growth	with *S. epidermidis*	SASDk78B	7	20	A1	−	−
6	Knee	n.d.	No	KPSSDk44A	14	20	A1	−	−
7	Knee	n.d.	No	KPSSDk45A	21	90	A1	−	−
8	Shoulder	No growth	No	SASDk4A	6	20	A1	+	K1 > L1 > H1 > A1
**Table 2B: Group 2: Infection likely**									
9	Shoulder	*C. acnes* (4/5)	with *C. namnatense*	SASDk73A/SASDk73D	7	100	A1	+	A1 > D1 > K7 > E3 > C1
				SASDk73C	7	20	K7		
10	Elbow	*C. acnes* (2/5)/*S. epidermidis* (2/5)	No	EASDk81A	3	>250	H1	+	K8 > H1 > K1
				EASDk81B/ EASDk81D/EASDk81E	3	>250	K8		
				EASDk81C	3	>250	K1		
11	Shoulder	n.d.	No	SPSSDk90A	4	>250	H1	+	D1 > H1 > K1 > A1 > E3
				SPSSDk90B/SPSSDk90C	4	>250	D1		
12	Shoulder	*C. acnes* (5/5)	No	SASDk69A	14	20	H1	+	H1 > D1 > A1
13	Shoulder	*C. acnes* (4/5)	No	SASDk57A/ SASDk57B/SASDk57C	3	100	K30	+	K30 > D1 > A1
**Table 2C: Group 3: Undetermined**									
14	Shoulder	*C. acnes* (2/5)	No	SASDk24A/SASDk24B	7	60	H1	−	−
15	n.d.	n.d.	No	PSSDk50A/PSSDk50D	7	50	K2	+	K2 > D1 > K1 > H1
				PSSDk50B/PSSDk50F	7	40	D1		
				PSSDk50C/PSSDk50E	7	20	K1		
16	Shoulder	No growth	No	SPSSDk64A	3	10	F26	+	F26
17	Elbow	n.d.	No	EPSSDk41A/EPSSDk41B	7	20	K1	+	K1 > D1 > H1
				EPSSDk41C	21	170	A1		

#### Group 1: Infection Unlikely

Eight implants were assigned to this group, including seven implants that were SF culture-positive but SLST PCR-negative (Table 2A). This indicated contamination after sonication, likely during the cultivation or incubation step. This group also contained the implants from which C2- (implant no. 1 and 2) and A1- (implant no. 5, 6, 7 and 8) type *C. acnes* strains were isolated; these were identified as clonal by WGS and subsequent genome comparison. It is likely that these contaminant strains originated from a single source, possibly from a source in the laboratory. In addition, SF specimens in this group had CFU counts that were below the EBJIS cut-off, or the *C. acnes* isolates took more than 14 days to grow. None of the patients had clinical features of OIAI, and tissue culture (if available) did not show any bacterial growth.

#### Group 2: Infection Likely

This group contained five implants, all of which were SF culture- and SLST PCR-positive (Table 2B). Three of them (implant no. 9, 10, and 13) had significant amounts of *C. acnes* in SF culture that grew within 7 days, as well as growth of *C. acnes* in tissue culture. Implant no. 12 had low growth of *C. acnes* in SF culture, but *C. acnes* was isolated from all five tissue biopsies. Regarding implant no. 11, tissue samples were not sent for culture, but SF culture revealed significant amounts of *C. acnes* SLST types D1 and H1, both of which were also detected in aNGS (relative abundance: D1, 69% and H1, 13%).

#### Group 3: Undetermined

Four implants could not be clearly classified in either the “infection unlikely” or “infection likely” group (Table 2C). Three of them were considered possible infections (implant no. 14, 15, and 16). Tissue and SF cultures were positive for *C. acnes* in implant no. 14, but the SLST PCR was negative. This could potentially be due to a false negative result in the SLST PCR. Implant no. 15 had low growth of three SLST types of *C. acnes*, all of which were also detected by aNGS. However, no tissue biopsies were sent for culture. Implant no. 16 was a suspected case of OIAI, but none of the tissue cultures showed growth of *C. acnes*. SF culture showed low growth of *C. acnes* SLST type F26 and aNGS also showed the presence of F26 as the sole SLST type. Implant no. 17 was considered as a possible contamination. SF culture of implant no. 17 revealed two SLST types, K1 and A1. Only A1-type *C. acnes* showed significant growth, but it was not detected by aNGS, suggesting that this A1 strain was likely a contaminant.

## Discussion

The increasing awareness of OIAIs caused by *C. acnes* has fueled debate of whether *C. acnes* isolation from orthopedic implants and periprosthetic tissues represents true infection or contamination or commensal colonization (Hudek et al., 2014, 2021; Patel et al., 2020; Dorrestijn and Pruijn, 2021; Torrens et al., 2022). Here, we applied an aNGS approach that, in combination with other clinical and laboratory investigations, can potentially help to differentiate between *C. acnes* infection and contamination in cases of suspected OIAI.

In the current study, we included 100 implants, irrespective of the reason for implant removal. This was done to minimize the chance of missing any potential *C. acnes* infection that does not present with (classical) features of OIAI. Differentiation of *C. acnes* OIAI from non-infectious causes of implant failure based on clinical symptoms alone is difficult, as they often present with non-specific clinical symptoms like joint stiffness or pain (Achermann et al., 2014; Renz et al., 2018a).

The cultivation time has previously been stated as a criterion to differentiate *C. acnes* contaminants from infection-causing isolates (Butler-Wu et al., 2011; Frangiamore et al., 2015; El Sayed et al., 2021). In the culture-dependent part of our study, agar plates were incubated for up to 28 days. *Cutibacterium acnes* isolates from all five cases of the “infection likely” group as well as the three possible infection cases of the “undetermined” group grew within 7 days, with one exception. However, four of the eight *C. acnes* isolates from cases of the “infection unlikely” group took more than 13 days to grow. This is in agreement with the results of previous studies that used thresholds of 11 and 13 days, respectively: it was observed that all *C. acnes* true-positives grew within these time periods; in contrast, 21.7–44% of contaminants grew beyond the cut-off time limits (Butler-Wu et al., 2011; Frangiamore et al., 2015).

The CFU count of bacteria isolated from SF is another important criterion used to determine the clinical relevance of the bacterial isolate. While previous studies have used different cut-offs, the latest EBJIS guidelines suggested a threshold of >200 or >50 CFU/ml from centrifuged and uncentrifuged SF, respectively (McNally et al., 2021). However, these thresholds may not be applicable concerning SGAB like *C. acnes*. Two of the five cases in the “infection likely” as well as all three possible infection cases of the “undetermined” group were associated with CFU counts below the recommended thresholds, while two out of eight cases of the “infection unlikely” group showed growth above the CFU count thresholds. This suggests that the CFU count is not a reliable criterion regarding SGAB like *C. acnes*.

Several previous studies have noted that *C. acnes* phylotypes IB (SLST type H) and II (SLST type K) tend to be more predominant in OIAIs (Sampedro et al., 2009; McDowell et al., 2013; Aubin et al., 2017; Lee et al., 2020; Salar-Vidal et al., 2021). The present study shows similar results. Concerning the culture-dependent results, all five implants in the “infection likely” group showed growth of either *C. acnes* phylotypes IB or II, and one implant showing growth of both phylotypes. In contrast, there was a predominance of phylotype IA_1_ *C. acnes* in the cases of the “infection unlikely” group: six out of eight cases showed only growth of A1- and C2-type *C. acnes* strains. A similar result was also found by the aNGS analysis: among all identified *C. acnes* types the relative abundances are in average highest for K- and H-type *C. acnes* in the 10 SLST PCR-positive cases (Figure 3C). Moreover, aNGS identified K- and/or H-type *C. acnes* in all but one of the 10 SLST PCR-positive cases. In seven cases, K- or H-type *C. acnes* were the dominating types in terms of relative abundance. This could suggest that strains of phylotypes IB and II have a higher probability to cause OIAI, whereas strains belonging to phylotype IA_1_ (with the possible exception of D1-type *C. acnes*) are more likely to represent contaminants or, possibly, non-involved commensals. However, it remains to be experimentally proven if strains of phylotypes IB and II have a higher OIAI-causing potential. Moreover, the association of type IB/II phylotypes with OIAI might not be so evident, as a few studies have found conflicting results, and reported a predominance of phylotype IA_1_ in OIAI (El Sayed et al., 2019; Liew-Littorin et al., 2019). It should be noted here that previous studies almost exclusively applied phylotyping to *C. acnes* isolates obtained by culture-dependent methods. Thus, additional studies, especially those based on culture-independent analyses of *C. acnes* phylotypes, are needed before it can be evaluated if phylotyping is a useful and reliable method to differentiate infectious strains of *C. acnes* from contaminants. Another interesting result from the current study was the finding that *C. acnes* OIAI appears to primarily be a heterotypic infection, i.e., an infection that is caused by more than one phylotype/SLST type of *C. acnes*. In many cases of the “infection likely” and “undetermined” groups multiple *C. acnes* types were found. Out of the nine implants in these two groups, five had heterotypic growth on culture, while aNGS detected heterotypic *C. acnes* populations in eight cases. As previously reported, *C. acnes* strains belonging to different SLST types can show similar colony morphologies (Bumgarner et al., 2020). Thus, heterotypic *C. acnes* infections could easily be missed in routine clinical diagnosis based on culture-dependent methods alone, where single colonies are randomly selected for further analysis. Interestingly, a study has reported that mono- and heterotypic *C. acnes* OIAIs appear to be two different clinical entities with different clinical histories and immune responses (El Sayed et al., 2019). While current treatment for both types of infection is the same, it could potentially differ in the future.

The present study also shows that several *C. acnes* strains isolated from SF culture were contaminants. *Cutibacterium acnes* detected in clinical samples could originate from the patients’ own skin during surgical incision, as the bacteria have been proven to persist on skin, despite standard surgical skin preparation (Lee et al., 2014; Hsu et al., 2020b). It could also originate from the OR, as *C. acnes* has been detected by culture from swabs exposed to air in the OR (Namdari et al., 2020a). Interestingly, regarding the 17 samples that exhibited growth of *C. acnes* in SF culture, all samples derived from hip (*n* = 2) and knee (*n* = 3) specimens were SLST PCR-negative cases and assigned to the “infection unlikely” group. This could indicate a higher risk of culture-dependent contamination associated with hip and knee surgery compared to shoulder and elbow surgery. Contamination may also occur in the laboratory, despite adequate precautions as demonstrated in this present study. The high risk of contamination due to the ubiquity of this microorganism needs to be carefully considered when interpreting positive *C. acnes* cultures.

Our study has a few important limitations. First, the study included a limited number of implants, with only 17 being culture-positive for *C. acnes.* This was due to the prospective nature of the study. Secondly, histopathology to detect the presence of neutrophils, which is an important criterion for diagnosis of OIAI is not done in Denmark and hence could not be included in the final evaluation. Thirdly, intraoperative tissue biopsy specimens were only sent for culture in 70.6% (12 of 17) of the cases; moreover, *C. acnes* isolates from tissue culture were not available for further analyses, e.g., for comparison with the *C. acnes* strains cultured from SF.

Other culture-independent methods could be used in future studies to support the diagnosis of *C. acnes* OIAI, including multiplex PCR and species- or even phylotype-specific PCR approaches (Sampedro et al., 2010; Morgenstern et al., 2018; Renz et al., 2018b; Sigmund et al., 2019). Metagenomic NGS (mNGS), which is an untargeted approach unlike aNGS, has recently been applied. While some studies (Weaver et al., 2019) showed that mNGS was superior to standard culture, especially in polymicrobial infections, others have shown poor correlation between mNGS and culture (Namdari et al., 2020b). In summary, we applied an aNGS approach to identify all SLST types/phylotypes of *C. acnes* in SF specimens and determine their relative abundances, with the aim to distinguish contaminant strains of *C. acnes* from OIAI-causing isolates. Our study showed the advantage of using a combination of clinical, laboratory, and microbiological methods, including culture-dependent and culture-independent analyses. An important finding was that not all *C. acnes* culture-positive cases represented true infections. This can potentially prevent overdiagnosis and unnecessary antibiotic treatment for the patients. However, additional studies, including the use of aNGS on tissue biopsy samples, are needed before the conundrum of *C. acnes* in OIAI can be entirely resolved.

## Data Availability Statement

The datasets presented in this study can be found in online repositories. The names of the repository/repositories and accession number(s) can be found at: https://www.ncbi.nlm.nih.gov/bioproject/PRJNA769547 and https://www.ncbi.nlm.nih.gov/bioproject/PRJNA801471.

## Ethics Statement

The studies involving human participants were reviewed and approved by Ethical Committee Region Midtjylland, Denmark. The patients/participants provided their written informed consent to participate in this study.

## Author Contributions

DSP, JL, TF-J, NPJ, CR, and HB contributed to the conception and design of the study. DSP performed wet lab benchwork and analyzed the data. AP and HB contributed to sequence data generation and analyses. DSP, JL, and HB wrote the manuscript. All authors contributed to the article and approved the submitted version.

## Funding

This research was supported with funds from the A. P. Møller foundation (no. 30903) for running costs and the “Fabrikant Vilhelm Pedersen og Hustrus Legat” (by the recommendation from the Novo Nordisk Foundation) for running costs and personnel (no. 30658).

## Conflict of Interest

The authors declare that the research was conducted in the absence of any commercial or financial relationships that could be construed as a potential conflict of interest.

## Publisher’s Note

All claims expressed in this article are solely those of the authors and do not necessarily represent those of their affiliated organizations, or those of the publisher, the editors and the reviewers. Any product that may be evaluated in this article, or claim that may be made by its manufacturer, is not guaranteed or endorsed by the publisher.

## Supplementary Material

Supplementary Figure S1 Implant characteristics and patient data. **(A)** Around 100 implants were processed in this study, including different types of implants from different locations. **(B)** Demographic and clinical data of 85 patients are listed.

Supplementary Figure S2 High-resolution figure of the core genome-based phylogeny of *C. acnes* obtained in this study. The core genome-based phylogeny of *C. acnes* is shown, using all genomes available at GenBank (*n* = 286; status June 2021) and the 36 *C. acnes* genomes sequenced here (in red).

## Abbreviations

aNGS: amplicon-based next-generation sequencing
CFU: colony-forming unit
OIAI: orthopedic implant-associated infection
MLST: multi-locus sequence typing
OR: operating room
PJI: prosthetic joint infection
SF: sonication fluid
SGAB: slow-growing Gram-positive anaerobic bacterium
SLST: single-locus sequence typing
SNV: single-nucleotide variant
WGS: whole genome sequencing
