# Supplementary figures and images for: Amplicon-Based Next-Generation Sequencing as a Diagnostic Tool for the Detection of Phylotypes of Cutibacterium acnes in Orthopedic Implant-Associated Infections
Source: Front Microbiol. 2022 Apr 7;13:866893. doi: 10.3389/fmicb.2022.866893 (PMC9022064; doi:10.3389/fmicb.2022.866893)

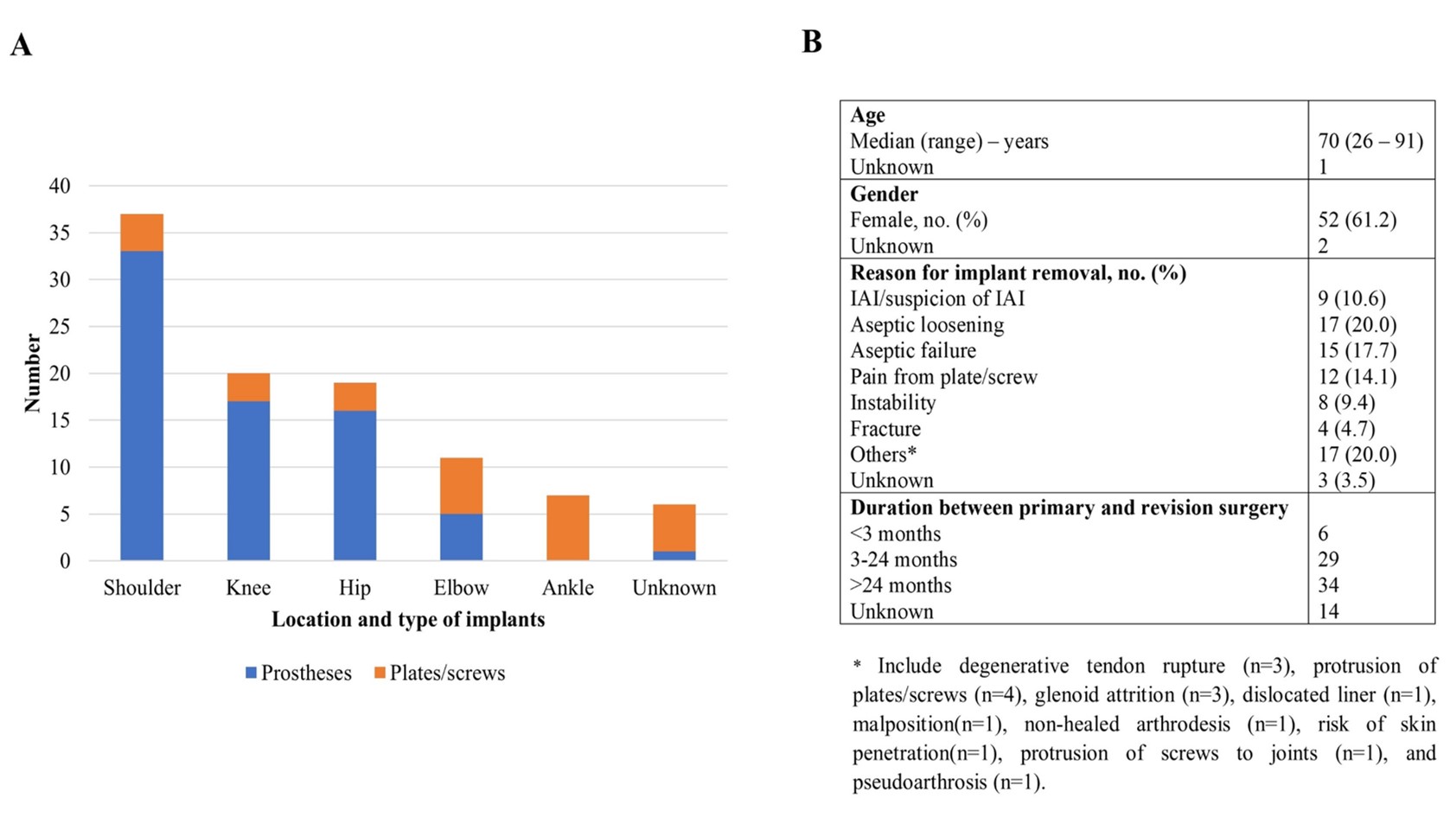

Supplement: Supplementary Figure S1 — Implant characteristics and patient data. (A) Around 100 implants were processed in this study, including different types of implants from different locations. (B) Demographic and clinical data of 85 patients are listed. [file Image_1.JPEG]

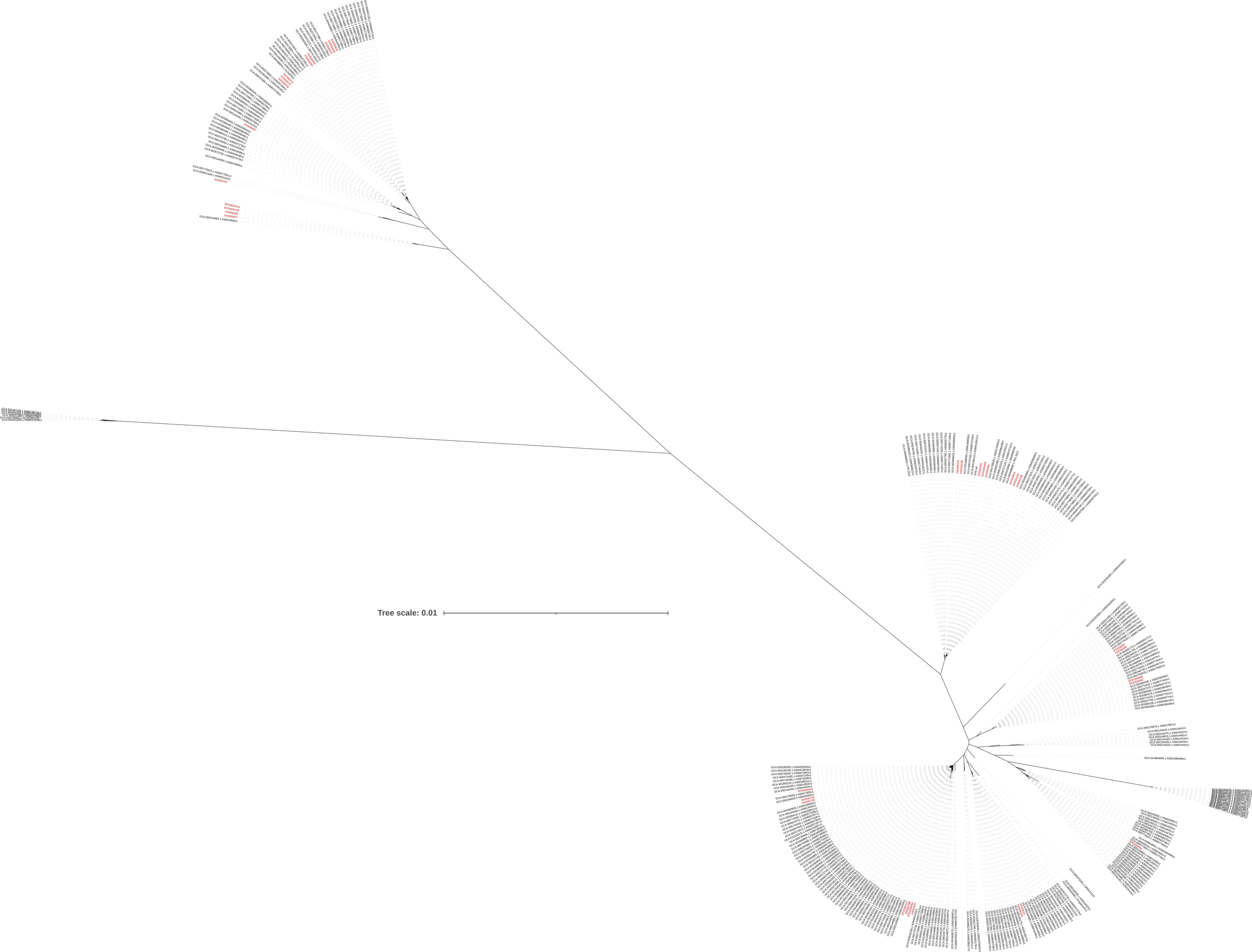

Supplement: Supplementary Figure S2 — High-resolution figure of the core genome-based phylogeny of C. acnes obtained in this study. The core genome-based phylogeny of C. acnes is shown, using all genomes available at GenBank (n = 286; status June 2021) and the 36 C. acnes genomes sequenced here (in red). [file Image_2.PDF]
