# Supplementary material for: Amplicon-Based Next-Generation Sequencing as a Diagnostic Tool for the Detection of Phylotypes of Cutibacterium acnes in Orthopedic Implant-Associated Infections
Source: Front Microbiol. 2022 Apr 7;13:866893. doi: 10.3389/fmicb.2022.866893 (PMC9022064; doi:10.3389/fmicb.2022.866893)
Supplement: Supplementary file 3 [file Table_1.DOCX]

**Table S1**: List of bacterial species identified by 16S rRNA gene sequencing

| **Bacteria** | **Number of positive SF culture**  **Significant growth*** | **Number of positive SF culture**  **Low growth*** | **Total** |
| --- | --- | --- | --- |
| **Aerobic bacteria** | | | |
| *Staphylococcus aureus*  *Staphylococcus epidermidis*  Coagulase negative staphylococci**  *Pseudomonas aeruginosa*  *Bacillus wiedemannii*  *Streptococcus mitis*  *Streptococcus mutans* | 6  4  4  1  1  1  1 | 1  3  3  0  0  0  0 | 7  7  7  1  1  1  1 |
| **Anaerobic bacteria** | | | |
| *Cutibacterium acnes*  *Cutibacterium avidum*  *Finegoldia magna*  *Propionibacterium namnatense*  *Bacteroides fragilis* | 13  1  1  0  1 | 8  0  0  1  0 | 21  1  1  1  1 |

^*^ the latest EBJIS guidelines for sonication fluid were applied (21)

^**^ except *Staphylococcus epidermidis*
