# Supplementary material for: Amplicon-Based Next-Generation Sequencing as a Diagnostic Tool for the Detection of Phylotypes of Cutibacterium acnes in Orthopedic Implant-Associated Infections
Source: Front Microbiol. 2022 Apr 7;13:866893. doi: 10.3389/fmicb.2022.866893 (PMC9022064; doi:10.3389/fmicb.2022.866893)
Supplement: Supplementary file 4 [file Table_2.DOCX]

**Table S2**: Details of the 36 *C. acnes* genomes obtained in this study

| **Strain** | **GenBank accession number** | **Phylotype** | **SLST**  **type** | **Size (bp)** | **Coverage**  **(fold)** | **Contigs**  **(#)** |
| --- | --- | --- | --- | --- | --- | --- |
| HASDk1A | JAJCWX000000000 | IA_1_ | C2 | 2537252 | 225 | 26 |
| SASDk4A | JAJCWW000000000 | IA_1_ | A1 | 2538553 | 155 | 22 |
| KASDk20A | JAJCWV000000000 | IA_1_ | C2 | 2538107 | 142 | 22 |
| HASDk23A | JAJCWU000000000 | IB | H14 | 2556466 | 199 | 30 |
| HASDk23B | JAJCWT000000000 | IB | H14 | 2548208 | 211 | 20 |
| SASDk24A | JAJCWS000000000 | IB | H1 | 2551664 | 216 | 24 |
| SASDk24B | JAJCWR000000000 | IB | H1 | 2575559 | 179 | 46 |
| SASDk40A | JAJCWQ000000000 | IB | H1 | 2554030 | 114 | 22 |
| EPSSDk41A | JAJCWP000000000 | II | K1 | 2466369 | 47 | 25 |
| EPSSDk41B | JAJCWO000000000 | II | K1 | 2463165 | 31 | 18 |
| EPSSDk41C | JAJCWN000000000 | IA_1_ | A1 | 2539978 | 125 | 29 |
| KPSSDk44A | JAJCWM000000000 | IA_1_ | A1 | 2529036 | 143 | 23 |
| KPSSDk45A | JAJCWL000000000 | IA_1_ | A1 | 2541399 | 125 | 24 |
| PSSDk50A | JAJCWK000000000 | II | K2 | 2489784 | 156 | 8 |
| PSSDk50B | JAJCWJ000000000 | IA_1_ | D1 | 2548467 | 114 | 24 |
| PSSDk50C | JAJCWI000000000 | II | K1 | 2483714 | 66 | 10 |
| PSSDk50D | JAJCWH000000000 | II | K2 | 2489684 | 109 | 9 |
| PSSDk50E | JAJCWG000000000 | II | K1 | 2464895 | 68 | 11 |
| PSSDk50F | JAJCWF000000000 | IA_1_ | D1 | 2543095 | 139 | 14 |
| SASDk57A | JAJCWE000000000 | II | K30 | 2484823 | 150 | 9 |
| SASDk57B | JAJCWD000000000 | II | K30 | 2484796 | 179 | 9 |
| SASDk57C | JAJCWC000000000 | II | K30 | 2484826 | 142 | 8 |
| SPSSDk64A | JAJCWB000000000 | IA_1_ | F26 | 2479333 | 164 | 14 |
| SASDk69A | JAJCWA000000000 | IB | H1 | 2545443 | 153 | 17 |
| SASDk73A | JAJCVZ000000000 | IA_1_ | A1 | 2485610 | 131 | 13 |
| SASDk73C | JAJCVY000000000 | II | K7 | 2494357 | 109 | 8 |
| SASDk73D | JAJCVX000000000 | IA_1_ | A1 | 2485967 | 147 | 13 |
| SASDk78B | JAJCVW000000000 | IA_1_ | A1 | 2548897 | 119 | 42 |
| EASDk81A | JAJCVV000000000 | IB | H1 | 2545793 | 123 | 14 |
| EASDk81B | JAJCVU000000000 | II | K8 | 2500357 | 180 | 37 |
| EASDk81C | JAJCVT000000000 | II | K1 | 2468028 | 142 | 21 |
| EASDk81D | JAJCVS000000000 | II | K8 | 2489686 | 175 | 18 |
| EASDk81E | JAJCVR000000000 | II | K8 | 2483977 | 158 | 10 |
| SPSSDk90A | JAJCVQ000000000 | IB | H1 | 2544388 | 162 | 18 |
| SPSSDk90B | JAJCVP000000000 | IA_1_ | D1 | 2536089 | 87 | 16 |
| SPSSDk90C | JAJCVO000000000 | IA_1_ | D1 | 2668773 | 136 | 77 |
