# Supplementary material for: Amplicon-Based Next-Generation Sequencing as a Diagnostic Tool for the Detection of Phylotypes of Cutibacterium acnes in Orthopedic Implant-Associated Infections
Source: Front Microbiol. 2022 Apr 7;13:866893. doi: 10.3389/fmicb.2022.866893 (PMC9022064; doi:10.3389/fmicb.2022.866893)
Supplement: Supplementary file 6 [file Table_4.DOCX]

**Table S4**: Summary of the 17 implants that were culture-positive for *C. acnes*

| **No.** | **Joint** | **Type of implant** | **Reason for implant removal** | **Tissue culture results** | **Sonication fluid –**  **culture-dependent** | | | | | **Sonication fluid – culture-independent** | | **Summary of results** |
| --- | --- | --- | --- | --- | --- | --- | --- | --- | --- | --- | --- | --- |
|  |  |  |  |  | **Polymicrobial** | **Strain name** | **Days to growth** | **CFU/ml** | **SLST type** | **SLST PCR** | **Amplicon NGS** |  |
| **1** | Hip | Prosthesis | Aseptic loosening | No growth | With *S. capitis* | HASDk1A | 6 | 50 | C2 | Neg | - | Infection unlikely |
| **2** | Knee | Prosthesis | Aseptic failure | No growth | No | KASDk20A | 21 | >250 | C2 | Neg | - | Infection unlikely |
| **3** | Hip | Prosthesis | Aseptic failure | No growth | No | HASDk23A/ HASDk23B | 7 | 20 | H14 | Neg | - | Infection unlikely |
| **4** | Shoulder | Prosthesis | Instability | No growth | No | SASDk40A | 21 | 20 | H1 | Neg | - | Infection unlikely |
| **5** | Shoulder | Prosthesis | Aseptic loosening | No growth | With *S. epidermidis* | SASDk78B | 7 | 20 | A1 | Neg | - | Infection unlikely |
| **6** | Knee | Plates / screws | Pain (plates/ screws) | n.d. | No | KPSSDk44A | 14 | 20 | A1 | Neg | - | Infection unlikely |
| **7** | Knee | Plates / screws | Pain (plates/ screws) | n.d. | No | KPSSDk45A | 21 | 90 | A1 | Neg | - | Infection unlikely |
| **8** | Shoulder | Prosthesis | Instability | No growth | No | SASDk4A | 6 | 20 | A1 | Pos | K1>L1>H1>A1 | Infection unlikely |

| **9** | Shoulder | Prosthesis | OIAI/ Suspicion of OIAI | *C. acnes* (4/5) | With *C. namnetense* | SASDk73A/ SASDk73D | 7 | 100 | A1 | Pos | A1>D1>K7>E3>C1 | Infection likely |
| --- | --- | --- | --- | --- | --- | --- | --- | --- | --- | --- | --- | --- |
|  |  |  |  |  |  | SASDk73C | 7 | 20 | K7 |  |  |  |
| **10** | Elbow | Prosthesis | Aseptic loosening | *C. acnes* (2/5)/  *S. epi-dermidis* (2/5) | No | EASDk81A | 3 | >250 | H1 | Pos | K8>H1>K1 | Infection likely |
|  |  |  |  |  |  | EASDk81B, EASDk81D/ EASDk81E | 3 | >250 | K8 |  |  |  |
|  |  |  |  |  |  | EASDk81C | 3 | >250 | K1 |  |  |  |
| **11** | Shoulder | Plates / screws | Pain (plates/ screws) | n.d. | No | SPSSDk90A | 4 | >250 | H1 | Pos | D1>H1>K1>A1>E3 | Infection likely |
|  |  |  |  |  |  | SPSSDk90B/ SPSSDk90C | 4 | >250 | D1 |  |  |  |
| **12** | Shoulder | Prosthesis | Aseptic failure | *C. acnes* (5/5) | No | SASDk69A | 14 | 20 | H1 | Pos | H1>D1>A1 | Infection likely |
| **13** | Shoulder | Prosthesis | Aseptic loosening | *C. acnes* (4/5) | No | SASDk57A, SASDk57B/ SASDk57C | 3 | 100 | K30 | Pos | K30>D1  >A1 | Infection likely |

| **14** | Shoulder | Prosthesis | Aseptic failure | *C. acnes* (2/5) | No | SASDk24A/ SASDk24B | 7 | 60 | H1 | Neg | - | possible infection |
| --- | --- | --- | --- | --- | --- | --- | --- | --- | --- | --- | --- | --- |
| **15** | n.d. | Plates / screws | Pain (plates/ screws) | n.d. | No | PSSDk50A/ PSSDk50D | 7 | 50 | K2 | Pos | K2>D1>K1>H1 | possible infection |
|  |  |  |  |  |  | PSSDk50B/ PSSDk50F | 7 | 40 | D1 |  |  |  |
|  |  |  |  |  |  | PSSDk50C/  PSSDk50E | 7 | 20 | K1 |  |  |  |
| **16** | Shoulder | Plates / screws | OIAI/ Suspicion of OIAI | No growth | No | SPSSDk64A | 3 | 10 | F26 | Pos | F26 | possible infection |
| **17** | Elbow | Plates / screws | Pain (plates/ screws) | n.d. | No | EPSSDk41A/EPSSDk41B | 7 | 20 | K1 | Pos | K1>D1>H1 | possible contami-nation |
